# Breaking Through the Multi-Mesa-Channel Width Limited of Normally Off GaN HEMTs Through Modulation of the Via-Hole-Length

**DOI:** 10.1186/s11671-017-2189-3

**Published:** 2017-06-17

**Authors:** Cheng-Yen Chien, Wen-Hsin Wu, Yao-Hong You, Jun-Huei Lin, Chia-Yu Lee, Wen-Ching Hsu, Chieh-Hsiung Kuan, Ray-Ming Lin

**Affiliations:** 1 Graduate Institute of Electronic Engineering and Department of Electrical Engineering, National Taiwan University, Taipei, 10617 Taiwan, Republic of China; 2 Department of Electronic Engineering, Chang Gung University, Taoyuan, 333 Taiwan, Republic of China; 3 Department of Radiation Oncology, Chang Gung Memorial Hospital, Tao-Yuan, 333 Taiwan, Republic of China; 4 Sino American Silicon Products Incorporated, Hsinchu, 300 Taiwan

**Keywords:** GaN, Enhancement mode, High-electron-mobility transistor (HEMT), Surface pinning effect

## Abstract

We present new normally off GaN high-electron-mobility transistors (HEMTs) that overcome the typical limitations in multi-mesa-channel (MMC) width through modulation of the via-hole-length to regulate the charge neutrality screen effect. We have prepared enhancement-mode (E-mode) GaN HEMTs having widths of up to 300 nm, based on an enhanced surface pinning effect. E-mode GaN HEMTs having MMC structures and widths as well as via-hole-lengths of 100 nm/2 μm and 300 nm/6 μm, respectively, exhibited positive threshold voltages (*V*_th_) of 0.79 and 0.46 V, respectively. The on-resistances of the MMC and via-hole-length structures were lower than those of typical tri-gate nanoribbon GaN HEMTs. In addition, the devices not only achieved the E-mode but also improved the power performance of the GaN HEMTs and effectively mitigated the device thermal effect. We controlled the via-hole-length sidewall surface pinning effect to obtain the E-mode GaN HEMTs. Our findings suggest that via-hole-length normally off GaN HEMTs have great potential for use in next-generation power electronics.

## Background

Wide-bandgap III–V nitrides are promising semiconductor materials for frequency and voltage operation because of their excellent material properties, including large band gaps, high critical electric fields, high-saturation electron velocities, and high conductivities [1, 2]. Accordingly, they are widely used in various applications, including light emitting diodes (LED) and transistors [3]. Furthermore, aluminum gallium nitride/gallium nitride (AlGaN/GaN) heterostructures form two-dimensional electron gases (2DEGs) suitable for the development of high-performance devices, taking advantage of the spontaneous and piezoelectric polarization of III-nitride compounds [4–6]. The quantity of a 2DEG is influenced by the proportion of polarization-induced doping, which directly affects the device characteristics [7–9]. Although they have many attractive properties, AlGaN/GaN high-electron-mobility transistors (HEMTs) have not found universal utility because their electronic characteristics can require complex circuit configurations for digital, power, RF, and microwave circuit applications. Accordingly, normally off operation would be essential for any future III–V semiconductor devices [10, 11]. Although some special fabrication techniques have been tested (e.g., use of recessed gates [12–14], insertion of p-type capping layers under the gate [15, 16], tunnel junction structures [17], fluoride ion implantation into the barrier under the gate [18], and inclusion of thin AlGaN barrier layers with a special metal gate and rapid thermal annealing (RTA) treatment [19]), they can worsen device performance and cause stability issues through processing-induced material damage and increased thermal and electric field effects.

Alternatively, a team at Hokkaido University found that AlGaN/GaN HEMTs fabricated with fin-nanochannels exhibited a shift in the threshold voltage (*V*_th_) in the positive direction [20, 21]. A group at Soochow University reported that the value of *V*_th_ underwent a systematic positive shift when the nanochannel width was less than 90 nm [22]. Researchers at Kyungpook National University considered the partial strain relaxation of the channels’ sides to explain the behavior [23]. A team at the Massachusetts Institute of Technology simulated the threshold voltage after surface passivation of GaN-based HEMTs and determined that positive values occurred when the width of the channel was less than 100 nm [24], the result of sidewall effects and increased tensile stress that decreased the electron concentration in the channel. Fin-shaped structures not only shift the threshold voltage but also improve gate controllability, due to the 3-D structure, which induces on-state performance while improving the off-state characteristics. The normalized maximum drain current (*I*_D_/mm) in an AlGaN/GaN HEMT having a fin-shaped structure is higher than that in a corresponding planar structure [25]. Although these methods have been used to fabricate E-mode HEMTs, it remains very challenging to develop high-performance normally off GaN power transistors. First of all, the combination of a low on-resistance (*R*_on_) and a low device total power is to achieve when the width of the channel is limited to be less than 100 nm. Although the value of *R*_on_ of the channel can be decreased by shrinking the length of the normally off gate, controlling the off-state drain leakage current poses another challenge because the gate width influences the transconductance and gate leakage through polarization coulomb field scattering and gate leakage paths [26, 27]. Deposited films can be used as gate dielectrics to improve these issues [28].

In this letter, we describe a breakthrough in the width limitation of tri-gate channels and propose a method for modulating the via-hole-length of the channels. Our device achieved the E-mode with a MMC structure width of 300 nm and a via-hole-length of 6 μm and exhibited a threshold voltage of 0.46 V. This approach not only decreased the device on-resistance (*R*_on_) but also could mitigate the Joule heating effect. By combining a 3-D tri-gate with various channel widths and via-hole-lengths, we achieved normally off GaN HEMTs having positive values of *V*_th_ of 0.79 and 0.46 V when the channel widths/via-hole-lengths were 100 nm/2 μm and 300 nm/6 μm, respectively.

## Methods

**Fig. 1:**
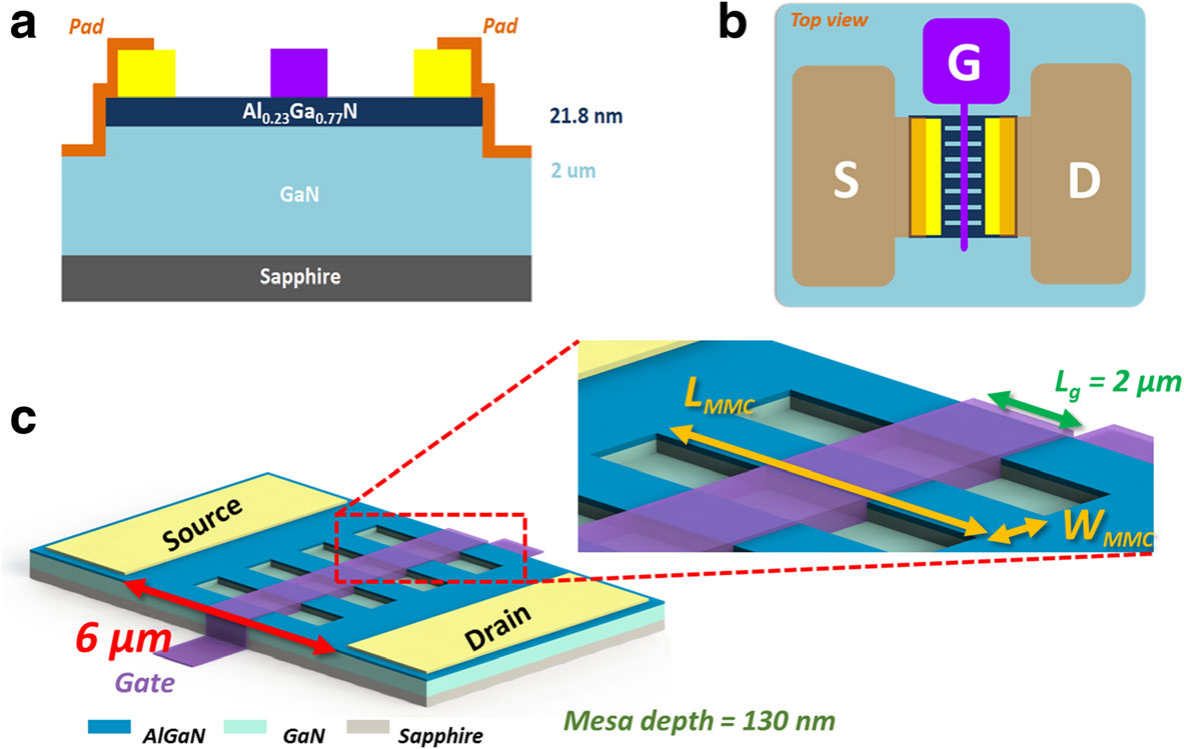
Schematic representations of **a** the cross-section of the HEMT structure, viewed from a direction parallel to the transistor channel; **b** the top-view of the HEMT structure; and **c** the 3-D structure of the HEMT

**Fig. 2:**
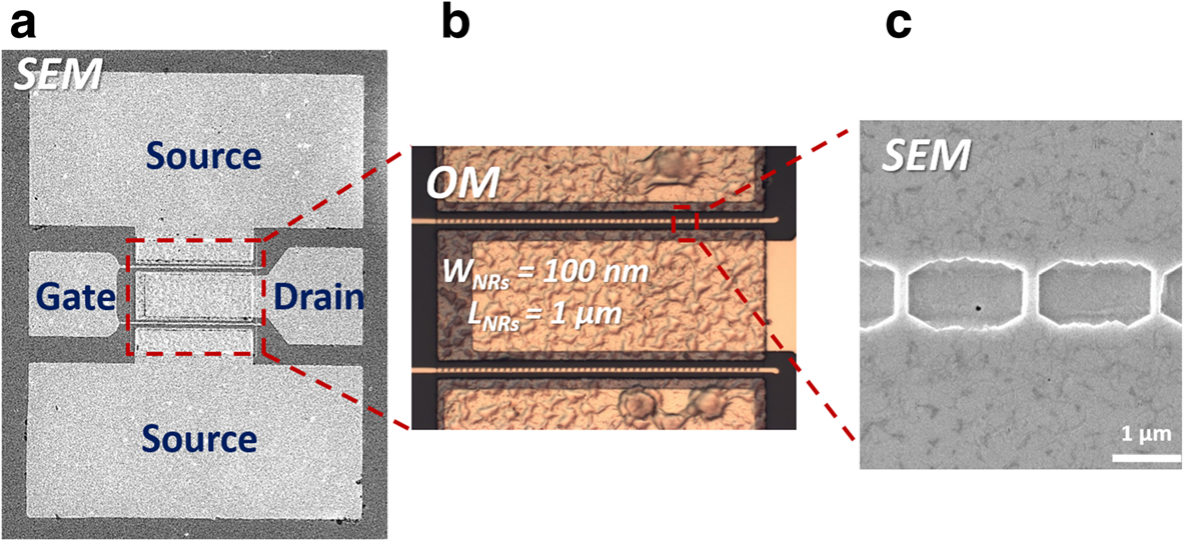
**a** Top-view SEM image of the device; **b** top-view OM image of the device, revealing a channel width and length of 100 nm and 1 μm, respectively; and **c** top-view SEM image of the channel

The AlGaN/GaN epi-wafer was grown on a (0001) sapphire substrate using a Nippon Sanso SR-2000 metal–organic chemical vapor deposition system (MOCVD). The growth of the epitaxial structure began with a GaN nucleation layer deposited at 600 °C. A 2-μm-thick unintentionally doped GaN buffer layer, a 21.8-nm-thick unintentionally doped AlGaN barrier layer with nominal 23% aluminum composition, and a 2-nm-thick GaN cap layer were then deposited at 1180 °C. Device processing was begun using an inductively coupled plasma (ICP) reactive ion etching (RIE) system with a BCl_3_/Cl_2_ gas mixture to isolate a 130-nm-deep mesa and etch a periodic trench structure. Subsequently, two processes were applied to restore the crystalline facets of the recess region and mesa sidewalls and decrease the levels of surface defects and ion bombardment damage. The first involved using molten KOH for crystallographic wet chemical etching to remove surface damage induced by dry etching and simultaneously produce smooth vertical sidewalls; the second involved applying piranha solution (a mixture of H_2_SO_4_ and H_2_O_2_) for surface cleaning and removal of organic residuals. Conventional photolithography with a mercury lamp was applied to define the drain, source, gate, and contact pads for DC measurements. Ohmic contacts to the AlGaN/GaN heterojunction, composed of titanium/aluminum/nickel/gold (Ti/Al/Ni/Au, 30/120/20/80 nm), were deposited onto the drain/source regions through electron beam evaporation and annealing at 850 °C for 30 s under vacuum. To complete the transistor channel, a gate electrode was fabricated through electron beam evaporation of Ni/Au (20/80 nm). Figure 1 provides schematic representations of the cross-section of the HEMT structure, a top view of the device, and a 3-D structural diagram of the device. The gate length (*L*_g_), MMC structure width (*W*_MMC_), MMC structure via-hole-length (*L*_MMC_), and MMC structure height (*H*_MMC_) were 2 μm, 100–500 nm, 1–6 μm, and 130 nm, respectively. Fins were connected in parallel. To enhance the surface pinning effect, the GaN HEMT via-hole-length structure was not subjected to passivation. Figure 2a presents a top-view scanning electron microscopy (SEM) image of the metallic surface in the source and drain region. The optical microscopy (OM) image in Fig. 2b reveals complete gates and channels; observing how many channels existed in the device was helpful when calculating the actual current. The surface appeared rugged in the image because, after annealing, the atoms migrated in the crystal lattice and the number of dislocations decreased, effectively decreasing the resistance. The SEM image in Fig. 2c confirmed the dimensions of the channel.

## Results and Discussion

To date, most technological developments in GaN high-voltage transistors have been based on AlGaN/GaN HEMTs, which are intrinsically depletion-mode (D-mode) devices because of the polarization-induced 2-D electron gas at the AlGaN–GaN interface [29]. Nevertheless, normally off GaN transistors will be required if the power electronics industry is to adopt GaN technologies widely.

**Fig. 3:**
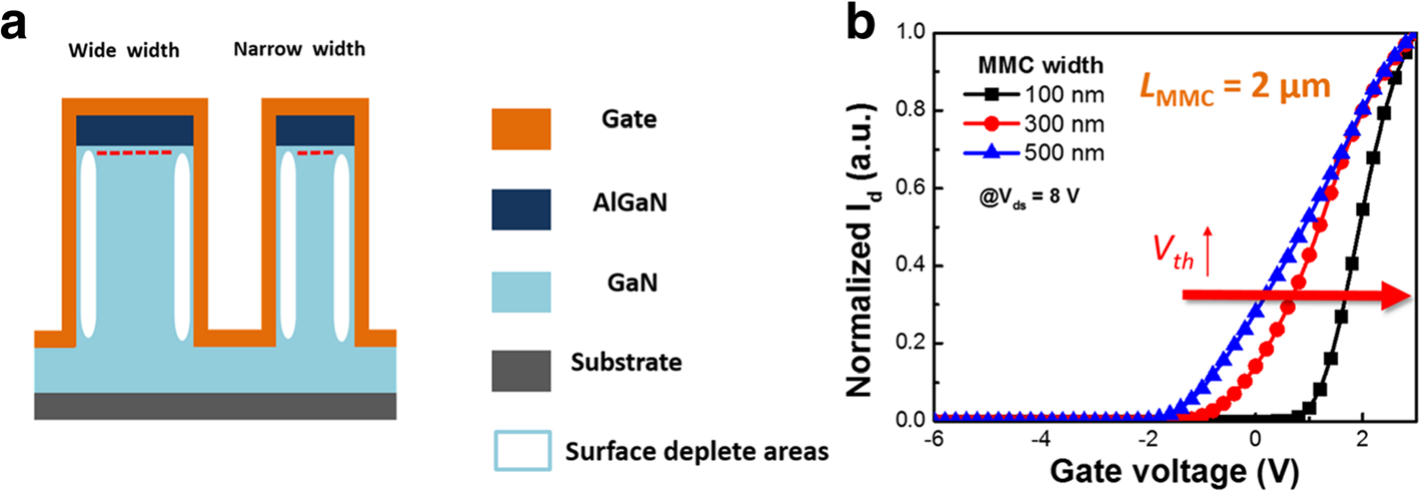
**a** Schematic representation of AlGaN/GaN HEMTs having wide and narrow channels. **b** *I*_DS_–*V*_G_ transfer characteristics measured for a device having a value of *L*_MMC_ of 2 μm and various values of *W*_MMC_

The number of dangling bonds on an (Al)GaN surface is approximately 10^15^ cm^−2^; these dangling bonds induce surface-depleted band bending as a result of a surface pinning effect. Figure 3a displays the lateral channel surface-depleted areas from the sidewall gates in the tri-gate structure. Researchers at Kyungpook National University reported a similar phenomenon [21]. Figure 3b presents the *I*_DS_–*V*_G_ transfer characteristics of devices having a fixed value of *L*_MMC_ of 2 μm and values of *W*_MMC_ of 100, 300, and 500 nm. When the drain-to-source voltage was 8 V, the values of *V*_th_ of these devices were +0.79, −1.32, and −2.18 V, respectively. Thus, a positive shift in the threshold voltage occurred as the channels became narrower. This phenomenon may have been due to lateral channel depletion and surface pinning of the 2-μm via-hole-length from the sidewall in the MMC via-hole-length structure through the effects of lateral channel depletion and via-hole-length surface bending.

**Fig. 4:**
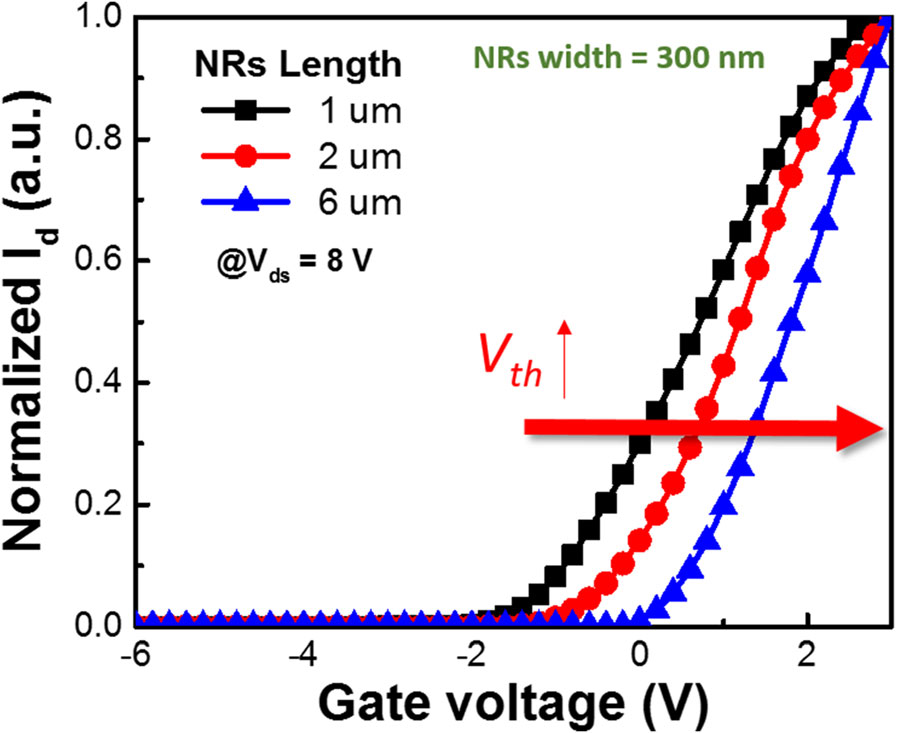
*I*_DS_–*V*_G_ transfer characteristics of devices having a fixed value of *W*_MMC_ of 300 nm and various values of *L*_MMC_

Figure 4 displays the I_DS_–V_G_ transfer characteristics of devices having a fixed value of *W*_MMC_ of 300 nm and values of *L*_MMC_ of 1, 2, and 6 μm. When the drain-to-source voltage was 8 V, the values of *V*_th_ were −2.12, −1.07, and +0.46 V, respectively. The device achieved normally off operation when the MMC length and width were 6 μm and 300 nm, respectively. Modulating the via-hole-length and channel width can provide a device displaying normally off operation. Table 1 lists the threshold voltages measured for various via-hole-lengths and multi-mesa-channel widths. When the channel width was fixed at 500 nm and the via-hole-length was increased from 0.8 to 6 μm, the value of *V*_th_ increased from −2.62 to −1.62 V, the saturation drain current decreased from 747 to 98 mA/mm, and the transconductance decreased from 270 to 40 mS/mm. When the channel width was fixed at 300 nm and the via-hole-length was increased from 0.8 to 6 μm, the value of *V*_th_ increased from −2.15 to +0.46 V, the saturation drain current decreased from 685 to 6.8 mA/mm, and the transconductance decreased from 290 to 7.4 mS/mm. When the channel width was fixed at 100 nm and the via-hole-length was increased from 0.8 to 2 μm, the value of *V*_th_ increased from −0.41 to +0.79 V, the saturation drain current decreased from 547 to 53 mA/mm, and the transconductance decreased from 400 to 67 mS/mm. The HEMT current handling capacity is strongly affected by the carrier concentrations [20, 21]. Accordingly, the devices’ saturation drain currents and transconductances were strongly affected by the side wall total surface states and the surface-depleted effect of the tri-gate channel upon varying the widths and via-hole-lengths of the GaN HEMTs. Compared with previously reported devices [23], our device has reached a new milestone for low-on-resistance, normally off GaN HEMTs.

**Table 1:** Threshold voltages of HEMTs having MMC structures of various lengths and widths, measured at a drain current of 1 mA/mm

MMC length	MMC width		
	100 nm	300 nm	500 nm
0.8 μm	-0.41 V	-2.15 V	-2.62 V
1 μm	-0.14 V	-2.12 V	-2.52 V
2 μm	+0.79 V	-1.32 V	-2.18 V
4 μm	–	-1.07 V	-2.07 V
6 μm	–	+0.46 V	-1.62 V

## Conclusions

We have prepared E-mode GaN HEMTs having a multi-mesa-channel (MMC) structure; they exhibited a positive threshold voltage of 0.46 V when the channel width and via-hole-length were 300 nm and 6 μm, respectively. We infer that the effects of both lateral channel depletion and via-hole-length surface bending. When containing a tri-gate having a MMC via-hole-length structure, the new normally off GaN HEMTs exhibited very low on-resistance, even when increasing the MMC structure width to 300 nm (formerly limited to less than 100 nm). In addition, modulation of the via-hole-length MMC structure provided normally off GaN HEMTs improving excellent power performance, as a result of increasing the MMC structure device width.

## References

[1] Chow TP and Tyagi R (1994) Wide bandgap compound semiconductors for superior high-voltage unipolar power devices. IEEE T Electron Dev 41:1481-1483.

[2] Davis RF (1991) III-V nitrides for electronic and optoelectronic applications. P IEEE 79:702-712.

[3] Abbasi MA, Ibupoto ZH, Hussain M, Nur O and Willander M (2013) The fabrication of white light-emitting diodes using the n-ZnO/NiO/p-GaN heterojunction with enhanced luminescence. Nanoscale Res Lett 8:320. 10.1186/1556-276X-8-320 PMC3711840 23849302

[4] Smorchkova IP, Elsass CR, Ibbetson JP, Vetury R, Heying B, Fini P, Haus E, DenBaars SP, Speck JS and Mishra UK (1999) Polarization-induced charge and electron mobility in AlGaN/GaN heterostructures grown by plasma-assisted molecular-beam epitaxy. J Appl Phys 86:4520-4526.

[5] Simon J, Protasenko V, Lian C, Xing H and Jena D (2010) Polarization-induced hole doping in wide–band-gap uniaxial semiconductor heterostructures. Science 327:60-64. 10.1126/science.1183226 20044569

[6] Guang HX, Gang ZD and Sheng JD (2015) Formation of two-dimensional electron gas at AlGaN/GaN heterostructure and the derivation of its sheet density expression. Chinese Phys B 24:067301.

[7] Li S, Ware M, Wu J, Minor P, Wang Z, Wu Z, Jiang Y and Salamo GJ (2012) Polarization induced pn-junction without dopant in graded AlGaN coherently strained on GaN. Applied Physics Letters 101:122103.

[8] Li S, Ware ME, Wu J, Kunets VP, Hawkridge M, Minor P, Wang Z, Wu Z, Jiang Y and Salamo GJ (2012) Polarization doping: Reservoir effects of the substrate in AlGaN graded layers. J Appl Phys 112:053711.

[9] Li S, Zhang T, Wu J, Yang Y, Wang Z, Wu Z, Chen Z and Jiang Y (2013) Polarization induced hole doping in graded Al_x_Ga_1-x_N (x=0.7 ~1) layer grown by molecular beam epitaxy. Appl Phys Lett 102:062108.

[10] Lu B, Matioli E and Palacios T (2012) Tri-gate normally-off GaN power MISFET. IEEE Electr Device L 33:360-362.

[11] Im KS, Kim RH, Kim KW, Kim DS, Lee CS, Cristoloveanu S and Lee JH (2013) Normally off single-nanoribbon Al_2_O_3_/GaN MISFET. IEEE Electr Device L 34:27-29.

[12] Im KS, Ha JB, Kim KW, Lee JS, Kim DS, Hahm SH and Lee JH (2010) Normally off GaN MOSFET based on AlGaN/GaN heterostructure with extremely high 2DEG density grown on silicon substrate. IEEE Electr Device L 31:192-194.

[13] Kim KW, Jung SD, Kim DS, Kang HS, Im KS, Oh JJ, Ha JB, Shin JK and Lee JH (2011) Effects of TMAH treatment on device performance of normally off Al_2_O_3_/GaN MOSFET. IEEE Electr Device L 32:1376-1378.

[14] Meneghini M, Scamperle M, Pavesi M, Manfredi M, Ueda T, Ishida H, Tanaka T, Ueda D, Meneghesso G and Zanoni E (2010) Electron and hole-related luminescence processes in gate injection transistors. Appl Phys Lett 97:033506.

[15] Uemoto Y, Hikita M, Ueno H, Matsuo H, Ishida H, Yanagihara M, Ueda T, Tanaka T and Ueda D (2007) Gate injection transistor (GIT)—A normally-off AlGaN/GaN power transistor using conductivity modulation. IEEE T. Electron Dev 54:3393-3399.

[16] Liu X, Zhan C, Chan KW, Owen MHS, Liu W, Chi DZ, Tan LS, Chen KJ and Yeo YC (2013) AlGaN/GaN metal–oxide–semiconductor high-electron-mobility transistors with a high breakdown voltage of 1400 V and a complementary metal–oxide-semiconductor compatible gold-free process. Jpn J Appl Phys 52:04CF06.

[17] Yuan L, Chen H and Chen KJ (2011) Normally off AlGaN/GaN metal–2DEG tunnel-junction field-effect transistors. IEEE Electr Device L 32:303-305.

[18] Wen Y, He Z, Li J, Luo R, Xiang P, Deng Q, Xu G, Shen Z, Wu Z, Zhang B, Jiang H, Wang G and Liu Y (2011) Enhancement-mode AlGaN/GaN heterostructure field effect transistors fabricated by selective area growth technique. Appl Phys Lett 98:072108.

[19] Endoh A, Yamashita Y, Ikeda K, Higashiwaki M, Hikosaka K, Matsui T, Hiyamizu S and Mimura T (2004) Non-Recessed-Gate Enhancement-Mode AlGaN/GaN High Electron Mobility Transistors with High RF Performance. Jpn J Appl Phys 43:2255-2258.

[20] Tamura T, Kotani J, Kasai S and Hashizume T (2008) Nearly temperature- independent saturation drain current in a multi-mesa-vhannel AlGaN/GaN high rlectron mobility transistor. Appl Phys Express 1:023001.

[21] Ohi K and Hashizume T (2009) Drain current stability and controllability of threshold voltage and subthreshold current in a multi-mesa-channel AlGaN/GaN high electron mobility transistor. Jpn J Appl Phys 48:081002.

[22] Liu S, Cai Y, Gu G, Wang J, Zeng C, Shi W, Feng Z, Qin H, Cheng Z, Chen KJ and Zhang B (2012) Enhancement-mode operation of nanochannel array (NCA) AlGaN/GaN HEMTs. IEEE Electr Device L 33:354-356.

[23] Im KS, Won CH, Jo YW, Lee JH, Bawedin M, Cristoloveanu S and Lee JH (2013) High-performance GaNbased nanochannel FinFETs with/without AlGaN/GaN heterostructure. IEEE T Electron Dev 60:3012-3018.

[24] Joglekar S, Azize M, Jones EJ, Piedra D, Gradecak S and Palacios T (2016) Impact of Al_2_O_3_ passivation on AlGaN/GaN nanoribbon high-electron-mobility transistors. IEEE T Electron Dev 63:318-325.

[25] Yeh PC, Lin YW, Huang YL, Hung JH, Lin BR, Yang L, Wu CH, Wu TK, Wu CH and Peng LH (2015) Threshold voltage controlled by gate area and gate recess in inverted trapezoidal trigate AlGaN/GaN MOS high-electronmobility transistors with photoenhanced chemical and plasma-enhanced atomic layer deposition oxides. Appl Phys Express 8:084101.

[26] Yang M, Lv Y, Feng Z, Lin W, Cui P, Liu Y, Fu C and Lin Z (2016) Study of gate width influence on extrinsic transconductance in AlGaN/GaN heterostructure field-effect transistors with polarization Coulomb field scattering. IEEE Trans Electron Devices 63:3908-3913.

[27] Kotani J, Yamada A, Ishiguro T, Tomabechi S and Nakamura N (2016) Direct observation of nanometer-scale gate leakage paths in AlGaN/GaN and InAlN/AlN/GaN HEMT structures. Phys Status Solidi A 213:883-888.

[28] Shih HY, Chu FC, Das A, Lee CY, Chen MJ and Lin RM (2016) Atomic layer deposition of gallium oixide films as gate dielectrics in AlGaN/GaN metal–oxide–semiconductor high-electron-mobility transistors. Nanoscale Res Lett 11:235. 10.1186/s11671-016-1448-z PMC4851678 27129687

[29] Ambacher O, Smart J, Shealy JR, Weimann NG, Chu K, Murphy M, Schaff WJ and Eastman LF (1999) Two-dimensional electron gases induced by spontaneous and piezoelectric polarization charges in N- and Ga-face AlGaN/GaN heterostructures. Jpn J Appl Phys 85:3222-3233.

